# Unpacking the buffering effect of social support figures: Social support attenuates fear acquisition

**DOI:** 10.1371/journal.pone.0175891

**Published:** 2017-05-02

**Authors:** Erica A. Hornstein, Naomi I. Eisenberger

**Affiliations:** Department of Psychology, Life Sciences, University of California, Los Angeles, Los Angeles, United States of America; Virginia Commonwealth University, UNITED STATES

## Abstract

Social support is associated with positive health outcomes, and research has demonstrated that the presence, or even just a reminder, of a social-support figure can reduce psychological and physiological responses to threats. However, the mechanisms underlying this effect are unclear, and no previous work has examined the impact of social support on basic fear learning processes, which have implications for threat responding. This study examined whether social support inhibits the formation of fear associations. After conducting a fear-conditioning procedure in which social-support stimuli were paired with conditional stimuli during fear acquisition, we found that the threat of shock was not associated with conditional stimuli paired with images of social-support figures, but was associated with stimuli paired with images of strangers. These findings indicate that social support prevents the formation of fear associations, reducing the amount of learned fears people acquire as they navigate the world, consequently reducing threat-related stress.

## Introduction

Research has consistently demonstrated a relationship between social support and positive health outcomes. It has been suggested that these health advantages arise, in part, because social support provides a buffer for individuals when dealing with life stress, and findings have shown that social support buffers against both the psychological and physiological threat response. Indeed, within the social buffering literature, it has been shown that individuals who have larger social networks, higher quality social relationships, and more access to social support resources have better physical and mental health, enjoying advantages ranging from a lower susceptibility to the common cold to a decreased risk of disease and death [1–3]. However, while this literature has established the impact of social support as a buffer, little prior work has examined the mechanisms whereby social support reduces physiological or psychological responses to threat. Consequently, the process by which social support provides this buffer remains not well understood. The present research seeks to explore this relationship by testing whether social support inhibits the formation of fear associations, consequently reducing fear responding and threat-related stress.

Evidence for this stress-buffering hypothesis can be found in both the animal and human literatures, and findings demonstrate that social support reduces both the psychological and physiological impact of threats. Animal research has shown that that the presence of familiar or close others decreases both the amount of escape and avoidance behavior exhibited in threatening contexts [4,5] decreases the amount of freezing behavior in response to a known threat [6], increases the ability to tolerate new environments [7,8], and decreases the amount of anxious behaviors exhibited following an experience of social defeat [9–11]. In addition to reducing behavioral and emotional stress responses, the presence of a familiar other can ameliorate physiological stress responses in the face of threatening events or situations. For example, the presence of a member of the same species with whom there is a bond reduces levels of cortisol when guinea pigs experience novel environments [12,13].

Consistent with the animal research on social buffering, work with humans has demonstrated that social support provides a similar buffering effect in threatening or stressful contexts. Findings show that perceptions of strong social support systems or relationships lead to reduced psychological stress in response to negative events [14–16]. Moreover, having higher levels of reported daily social support is correlated with reduced cortisol levels when faced with social stressors [17] as well as reduced heart rate and blood pressure in the face of acute stressors [18–20]. Additionally, it has been demonstrated that social support can provide a buffer for individuals by mitigating the experience of pain [21–23]. Recent work suggests that this pain-mitigating effect may be due to decreased activity in neural regions associated with the distressing aspect of pain and increased activity in neural regions associated with safety [24]. Altogether, these findings point to the important role played by social support in regulating stress in the face of threat, leading to lower behavioral and physiological reactivity, and possibly resulting in fewer negative downstream health consequences.

One possible mechanism by which social support provides this buffer against stress is by acting as a powerful natural safety signal—communicating protection and consequently reducing psychological and physiological threat responses. Indeed, recent research has shown that social-support figures are one category of prepared safety stimuli, less easily becoming associated with threat and reducing conditional fear responses, and that the presence of social-support figure reminders potentially leads to longer lasting fear extinction [25]. Thus, by signaling safety and interfering with normal fear learning processes, social support may reduce threat-related stress and increase positive health outcomes.

However, to date, no work has examined the effect of social support on the way fear is learned for other events or stimuli in the environment. It is possible that social support not only signals safety and reduces fear responding, but also decreases the amount of fear associations formed overall. Therefore, we designed a study to examine the impact of social support on fear learning, examining the effect of social-support-figure stimuli on the association of threat with other cues and testing whether social-support stimuli buffer individuals against acquiring new fears.

In order to test the impact of social support on fear learning, we used a fear-conditioning paradigm to examine whether the presence of social-support figure stimuli, defined here as the individual from whom a participant receives the most social support (in the form of care and resources) on a daily basis, reduced fear acquisition for a separate neutral cue. Specifically, we assessed conditional fear responses when a social-support figure’s image, or a stranger’s image, was paired with a neutral cue during fear acquisition. We hypothesized that while a conditional fear response would be acquired for neutral stimuli paired with images of strangers, no conditional fear response would be acquired for neutral stimuli paired with images of social-support figures.

## Methods

### Participants

Data were analyzed from a final sample of 20 participants (mean age = 19.70, 15 females) who completed the study procedures. This sample size was chosen based on a priori power analyses (see supplemental materials). In total, 30 participants were recruited, 2 participants were excluded based on the telephone screening, 4 were excluded based on the SCR screening, and 4 were excluded due equipment malfunction. All participants were recruited from the UCLA community and provided written consent. All consent and experimental procedures were approved by the UCLA IRB (#11–000896).

### Procedure

The study had three parts: telephone screening, pre-screening session in the lab, and experimental session. Participants first completed the telephone screening session and pre-screening session to determine if they were eligible to participate in the experimental session (see supplemental materials). During the pre-screening session, they were asked to select “the individual who gives you the most support on a daily basis” and were instructed that these individuals could come from any relationship (e.g. parent, friend, significant other). They then were asked to rate how much social support this individual gives everyday on a scale of 1–10 (mean rating = 8.60). They were then instructed to send a digital photograph of this individual to the experimenter before the experimental session.

For the experimental session, participants returned to the lab and first completed a shock calibration procedure in order to determine the level of shock to be used for each individual participant during the experiment, such that it was extremely uncomfortable, but not painful (see supplemental materials). Participants then underwent a fear-conditioning session with 2 sets of stimuli. Each set comprised 2 neutral images from one of two object categories (clocks, stools), with one image from each set becoming a CS+ and one becoming a CS-, and both being paired with the same secondary image (social-support figure, stranger) during the acquisition stage of the experiment. There were three stages of the experiment: Habituation, Acquisition, and Extinction. For each stage, images were presented for 6 s, followed by a 10-s inter-stimulus-interval in a pseudo-random presentation order that was counter-balanced across participants. Fear responses were evaluated using Skin Conductance Response (SCR) measurements.

During the Habituation stage of the experiment, participants saw 3 non-reinforced presentations of each neutral image. This was done in order to ensure that there were no pre-existing characteristics of either of the neutral stimuli in each set that might account for later differences in SCR, and none were found (ps>.195).

**Fig 1:**
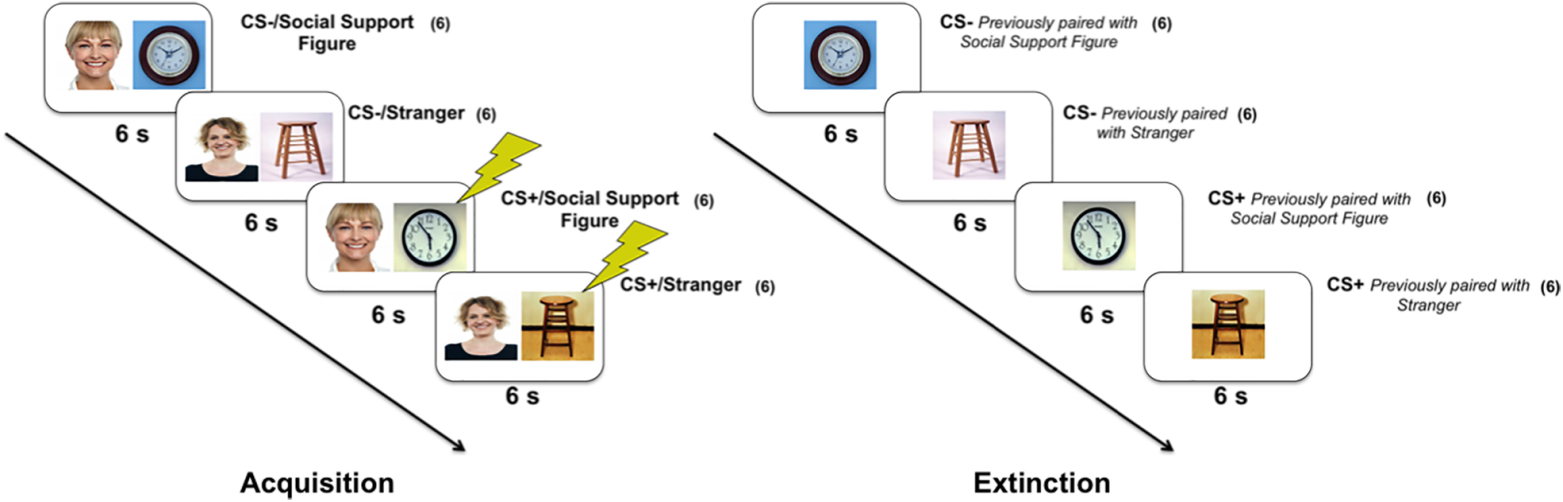
Acquisition and extinction procedures. Example of the CS/secondary-image and shock pairings presented during the acquisition stage of the experiment and CS alone presentations during the extinction stage of the experiment. During acquisition, participants viewed two sets of two neutral images (clocks, stools), and both images from each set were paired with the same secondary image (social support figure, stranger). One of these pairings from each set was paired with shock, the CS+/secondary-image pairing, and one pairing was never paired with shock, the CS-/secondary-image pairing. Following acquisition was an extinction stage, during which each neutral image was once again presented on its own (no secondary image and no shock). Conditional fear acquisition was measured by comparing SCR for the CS+/secondary-image pairing to the CS-/secondary-image pairing within each set of neutral images during the acquisition stage. The numbers in parentheses indicate number of CS/secondary-image or CS alone presentations. All presentations were 6s followed by a 10s ISI. The order for both stages was pseudo-randomized, and counterbalanced across participants.

Following this, there was the Acquisition stage (see Fig 1), during which participants viewed six presentations of the images from each set paired with one of two secondary images: the social-support figure image provided by the participants, or an image of a stranger that was gender-, age-, and ethnicity-matched to the social-support figure. One of the CS/secondary-image pairings from each set was consistently presented with a co-terminating 200ms electric shock (CS+/secondary-image pairing: 100% reinforcement schedule), while the other CS/secondary-image pairing was never paired with shock (CS-/secondary-image pairing). After the Acquisition stage, participants had a five minute long break during which they viewed a video clip about airplanes. Finally, during the Extinction stage, there were six non-reinforced presentations of each original neutral image once again presented alone, with the secondary image removed.

### Data analysis strategy

In order to examine fear learning patterns across conditions (social-support paired or stranger-paired), 2x2 repeated measures ANOVAs were run (paired image condition x reinforcement type) to examine mean SCR for the two CS+s and the two CS-s both during and post acquisition. If there was a significant interaction of paired image condition and reinforcement, it was considered that conditional fear was acquired differently for two conditions, and follow-up paired-sample t-tests were run to examine these differences.

In order to examine fear acquisition within each condition, paired-samples t-tests were run comparing acquisition means for the CS+/secondary-image pairing to the CS-/secondary-image pairing in the social-support-paired and stranger-paired conditions. If the SCR aroused by the CS+/secondary-image pairing was significantly higher than that of the CS-/secondary-image pairing, it was considered that a conditional fear response was acquired. Paired-samples t-tests were also run on the SCR aroused by the neutral images during the first trial of the extinction stage—the first trial after the secondary image had been removed and each neutral image was presented alone once again.

Additionally, we ran paired-samples t-tests to evaluate the effect of condition on fear acquisition, comparing mean difference scores (CS+/secondary-image vs. CS-/secondary-image) within each condition. Similarly, we ran paired-samples t-tests to evaluate fear responses post-acquisition, comparing SCR difference scores (CS+ vs. CS- from each condition) from the first trial of extinction.

## Results

In order to determine the effect of the presence of a social-support image during fear acquisition, we first examined the effect of paired image condition (social support or stranger) and reinforcement type (CS+ or CS-) on fear responding. We found there was a significant interaction of these factors during the acquisition stage, when the paired images are still on the screen, F(1,19) = 10.326, p = .005, η_p_^2^ = .352, as well as during the first trial post the acquisition stage, when the paired images have been removed, F(1,19) = 5.195, p = .034, η_p_^2^ = .215, indicating that there are differences in fear learning across conditions.

**Fig 2:**
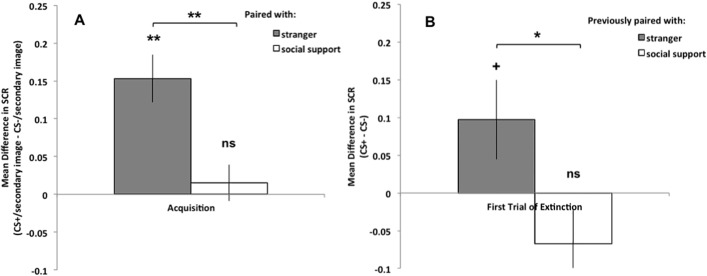
Conditional fear acquisition. A). SCR from the Acquisition stage: conditional fear responses were evaluated by comparing the CS+/secondary-image to the CS-/secondary-image from each condition (social-support-paired, stranger-paired). A conditional fear response was acquired in the stranger-paired condition, but not in the social-support-paired condition. B). SCR from the first trial of the Extinction stage: conditional fear responses were evaluated by comparing the CS+ and CS- from each condition when once again presented alone (with the social support or stranger image removed). A marginal conditional fear response was still present for the CS+ that had been paired with a stranger image, but not for the CS+ that had been paired with a social-support-figure image. All error bars indicate standard error. Asterisks indicate a statistically significant difference score (** indicates p< or = .001, * indicates p < .05), “+” indicates a marginal difference score (p < .1), and “ns” indicates a non-significant difference.

Next, we examined whether there was a difference in fear acquisition across paired image conditions. We evaluated fear acquisition for both the social-support-paired and the stranger-paired conditions, and found that while participants did acquire fear for CS+s paired with strangers, t(19) = 4.86,p < .001, 95% CI[0.09,0.22], they did not acquire fear for CS+s paired with social-support figures, t(19) = .626,p = .539, 95% CI[-0.03,0.06], (see Fig 2A). Further examination showed that the effect of condition on fear acquisition was significant, t(19) = -3.80,p = .001, 95% CI[-0.21,-0.06], such that fear acquisition in presence of a social-support figure image was significantly less than fear acquisition in the presence of a stranger image. Together, these results demonstrate that the presence of social-support stimuli inhibits fear acquisition for other cues, providing support for our hypotheses.

In addition, we found that even after the secondary images were removed, a marginal fear response was still present in the stranger-paired condition, t(19) = 1.84,p = .082, 95% CI[-0.01,0.21], but there was no fear response present in the social-support-paired condition, t(19) = -1.52,p = .144, 95% CI[-0.16,0.02] (see Fig 2B). While the fear response for the stranger condition during this stage is only marginal, likely due to the relatively weak fear conditioning manipulation used here, it is trending toward significant and indicates that the fear association for the CS+ in the stranger-paired condition lasted beyond the end of the fear acquisition stage and the removal of the stranger image. Moreover, examination across conditions revealed that the fear response was significantly less in the social-support-paired condition than in the stranger-paired condition, t(19) = -2.28,p < .05, 95% CI[-0.31,-0.01]. Future work must build on this exploratory study to more closely examine the lasting effects of the presence of social support figures during fear acquisition.

## Discussion

Social support has long been linked to positive health outcomes. One explanation for these health benefits is that social support buffers individuals against life stress, and it has been demonstrated that the presence of social-support reminders reduce both psychological and physiological responses to threat. However, no research to date has examined the relationship between social support and fear learning for other cues. In the current research, we examined whether social support not only signals safety and inhibits the fear response, but also reduces fear associations formed for other neutral cues. Results showed that the presence of social-support reminders inhibits the formation of fear associations. Specifically, we found that when an image of a social-support figure was paired with a neutral cue during fear acquisition, participants did not form a fear association for that cue, although they did form this association for a neutral cue paired with a stranger’s image.

Additional results showed that when presented alone after fear acquisition was completed, a marginal fear response remained for the neutral cue that had been paired with a stranger’s image, but there was none for the neutral cue that had been paired with a social-support figure’s image. Although these findings were only trending toward significant, they indicate that the benefits of social support continue even after an aversive event is over or a stressor is removed. This is interesting given that social integration, (participation in/a sense of belonging to a social network) has been shown to promote positive health outcomes even in the absence of current stress [for review, see: 26]. The current findings may give insight into the process underlying this effect—individuals with stronger social ties form fewer fear associations, while those who lack social ties form more fear associations, resulting in increased fear responding and stress as they interact with the world.

One possible alternative explanation for these findings is that the presence of stranger images augmented fear acquisition, as opposed to the presence of social support figure images reducing fear acquisition. However there are at least three reasons why this possibility seems unlikely. First, previous work using similar methods has demonstrated no difference in the safety or threat signaling function of images of strangers compared to images of neutral objects [25], indicating that stranger images would not be expected to have any impact on fear learning processes beyond that of neutral stimuli. Second, finding that the presence of stranger images augmented fear acquisition would imply that the fear conditioning procedure used here was not strong enough to produce fear acquisition except in the presence of strangers. Yet, similar fear conditioning procedures have been used in other studies by this team [25] and others [27–29], in which expected patterns of fear learning were produced, indicating that fear learning should occur under the current procedures. Finally, to the extent that the stranger faces were interpreted as threatening (though unlikely because all stranger stimuli were smiling faces which have been shown to be perceived as warm and approachable [30] and to yield reward-related neural activity [31]), this should actually lead to reduced fear acquisition to a separate conditional fear stimulus. Specifically, although fear acquisition is enhanced to threatening stimuli, the presence of a threatening stimulus in the context of learning fear to another cue actually prevents fear acquisition from occurring, a phenomenon known as blocking [32]. This is the opposite pattern of what we observed here. Thus, the results described here likely do not reflect an augmentation of fear acquisition caused by the presence of stranger stimuli, but rather a reduction of fear acquisition caused by the presence of social support stimuli.

This reduction in fear learning may stem from the ability of social-support stimuli to naturally, without any specific training, signal safety. It is possible that other characteristics of close others, such as being familiar or rewarding, could explain these effects. This is unlikely, however, given previous findings showing that while fear can be acquired for familiar or rewarding stimuli, it cannot be acquired for social-support stimuli [25]; nonetheless, future research is required to definitively rule out this possibility. Future work is also required to identify the boundaries of social support as a buffer against fear learning, such as investigating whether this effect is found when the conditional stimuli used are fear-relevant (e.g., prepared fear stimuli), or whether this effect is found in participants who are more prone to developing fears (e.g., anxious individuals). Similarly, while the current work could not address the role of gender in these effects, due to the limitation that data collected came from a sample that was 75% female, follow-up studies should investigate whether reduction of fear acquisition occurs equally across males and females.

In addition to exploring the boundaries of the buffering effects demonstrated here, future work must isolate the mechanism underlying the safety signaling properties of social support. While it is possible that social-support stimuli simply act as a buffer against pain, reducing the experience of shock, or increase feelings of safety in the moment, decreasing fear expression, a more likely explanation is that social-support stimuli alters the way in which fears are acquired. Social-support stimuli are known to trigger the release of endogenous opioids [for review, see: 33], which play a fundamental role in the error-correction process underlying fear learning [32,34], and therefore may disrupt the error-correction calculations that lead to fear acquisition. Further clarification of these mechanisms and effects will help develop a better understanding of how and when social support interferes with fear learning, bolstering positive health outcomes.

Altogether, these findings build on previous research demonstrating the buffering effects of social support and reveal a clearer picture of how social support might reduce psychological and physiological stress. By inhibiting the formation of fear associations for other cues, our close relationships may allow us to navigate the world with fewer learned fears, thus decreasing the activation of the threat response. Together with previous findings showing that social-support figures fulfill the requirements of prepared safety stimuli [25], these results suggest that social support may be helpful in preventing the formation of unnecessary or maladaptive fear associations and reducing threat related stress.

## Supporting information

S1 Text Unpacking the buffering effect of social support supporting materials. (DOCX)
