# Supplementary material for: Unpacking the buffering effect of social support figures: Social support attenuates fear acquisition
Source: PLoS One. 2017 May 2;12(5):e0175891. doi: 10.1371/journal.pone.0175891 (PMC5413011; doi:10.1371/journal.pone.0175891)
Supplement: S1 Text — (DOCX) [file pone.0175891.s001.docx]

**Unpacking the buffering effect of social support figures: Social support attenuates fear acquisition**

Hornstein EA & Eisenberger NI

Supporting Material

**Materials and Methods**

*Screening and Procedures*

**Telephone Screening Session.** After a telephone screening with the experimenter, participants were not allowed to participate if they were pregnant, had a history of mental illness, or were currently taking any mental health related medication.

**Pre-screening Session.** If they passed the telephone screening, participants were asked to come into the lab for a 30-minute pre-screening session. During this session, participants were tested to ensure that their Skin Conductance Response (SCR) could be detected by the equipment being used for the experiment. SCR for all participants was measured using the BioPac MP100 system with EDA Isotonic Gel Electrodes, and data were collected using AcqKnowledge 3.9 software (BioPac Systems, Inc., Aero Camino Goleta, CA). For the test, and the following experiment session, electrodes were placed on the palmar side of participants’ medial phalanges on the fore and middle fingers of the left hand. In order to activate the sympathetic nervous system and consequent increases in SCR, participants were asked to breathe in deeply, allowing the experimenter to monitor their responses and determine if an SCR increase was detected. If a participant’s response was not detected by the equipment, he or she was excluded from the experiment.

**Experiment Session.** At the beginning of the experiment session, an electric shock calibration procedure was conducted to determine the appropriate level of shock to be applied for each individual participant during the experiment. Electric shock was applied to participants via a bar lead electrode placed on the write wrist, and was delivered from a SD9 Pulse Stimulator from Grass Technologies (Natus Neurology, Inc. – Grass Products, Middleton, WI). For the shock calibration procedure, and during the experiment session, participants were exposed to a 200ms electric shock starting at 30 volts, and increasing in 5-volt increments. Participants were instructed to inform the experimenter when the shock became extremely uncomfortable, but was not yet painful, and that level of shock was then used during the experiment session. This work-up procedure was used previously [see: 1] and was designed based on work-up procedures from previous studies in which shock was used as an aversive stimulus during human fear conditioning [see: 2-4]. Average voltage (as decided upon by the experimenter and the participant during the shock calibration procedure) was 49.87 volts.

All stimuli were presented using E-Prime 2.0 software (Psychology Software Tools, Inc., Sharpsburg, PA).

*Data Analysis*

**Participants**. The target sample size for the experiment was n=20, therefore participants were recruited until the targeted sample size was achieved. Target sample size and exclusion criteria, as described above, were based on guidelines from previous human fear conditioning studies [see: 1, 2, 4]. Additionally, an a priori power analysis was conducted using results from a previous study conducted that used a similar fear conditioning procedure to assess fear acquisition for three different types of stimuli (in order to examine whether social support figures pass the retardation-of-acquisition test). Based on a 3x2 repeated measures ANOVA using SCR from the first trial following the fear acquisition procedure to examine fear acquisition (with a reasonably large effect size of η_p_^2^= .185), we used G*Power 3.1 to run a power analysis (repeated measures ANOVA with six measurements, power of .95, alpha of .05, effect size *f=*.48) and found that a sample size of n=12 is required to examine fear acquisition using this procedure. Therefore the selected sample size of n=20 is well within the range required to conduct the following analyses.

**Pre-processing.** All SCR data were collected and pre-processed using AcqKnowledge 3.9. Data were pre-processed using a low pass filter and smoothed, and then evaluated using peak-to-peak analysis for each trial (each image/paired images presentation). The peak-to-peak amplitude was measured in micro-siemens (μS) for the first response that occurred between .5s-4.5s after stimulus onset [these methods were chosen based on previous SCR analysis recommendations, see: 5]. All measurements were then normalized using a square-root transformation.

During any trial, if there was no peak (no rise in SCR) during the .5-4.5s stimulus window, the trial was scored as a zero response trial. In addition, a .02 μS threshold was used for peak-to-peak amplitudes, thus if a peak-to-peak amplitude for any trial was below 02 μS, the trial was scored as a zero response trial. Finally, if the participant breathed deeply or moved during a trial, as recorded by the experimenter during the experiment session, the trial was excluded from data analysis.

**Scoring**. The habituation mean was calculated by averaging across all habituation trials. The acquisition mean was calculated by averaging across the final four trials of the acquisition stage for each stimulus. For each participant, if the acquisition mean was not greater for the CS+ than the CS- (CS+ - CS- > 0) or if there were no peaks during any of the trials, it was considered that no conditional fear had been acquired for that condition. After acquisition, once the secondary image was removed and the original neutral images were presented alone once again, conditional fear response for each stimulus was compared using the first trial of the extinction stage for each stimulus type.

**Additional Analyses and Data**

*Habitation*

We found no differences between the stimuli in each set (clocks, stools): comparing the future CS+ to the future CS- in the social-support paired condition, t(19)=-0.77,*p*=.451, 95% CI[-0.07,0.03], and the stranger paired condition, t(19)=1.34,*p*=.195), 95% CI[-0.01,0.05].

*Trial-by-trial SCR*

**Acquisition Stage**

**
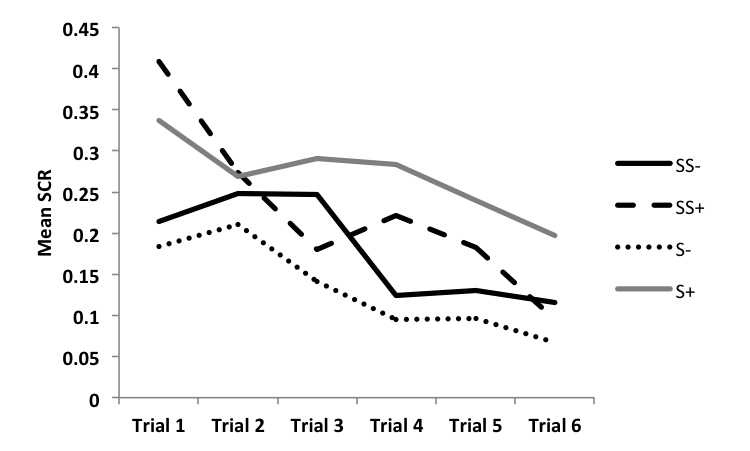
**

**Extinction Stage**

**
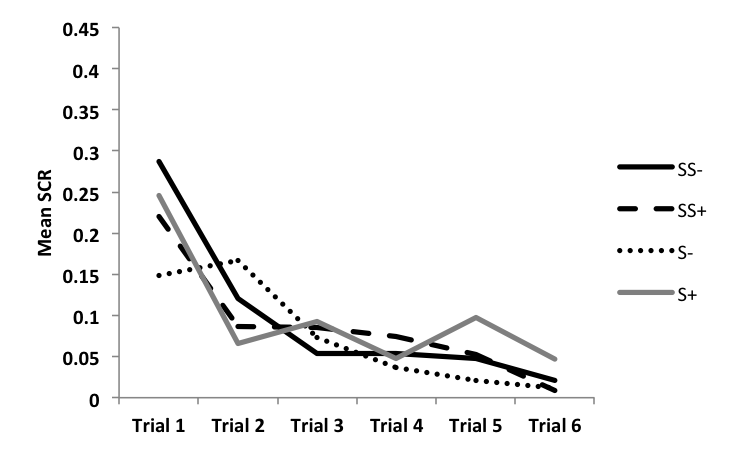
**

**References**

1. Hornstein EA, Fanselow MS, Eisenberger NI. A safe haven: Social-support figures are prepared safety stimuli. Psychological Science 2016; doi:10.1177/0956797616646580.
2. Olsson A, Ebert JP, Banaji MR, Phelps EA. The role of social groups in the persistence of learned fear. Science 2005; 309: 785-787.
3. Phelps EA, Delgado MR, Nearing KI, LeDoux JE. Extinction learning in humans: Role of the Amygdala and vmPFC. Neuron 2004; 43: 897-905.
4. Schiller D, Monfils M, Raio CM, Johnson DC, LeDoux JE, Phelps EA. Preventing the return of fear in humans using reconsolidation update mechanisms. Nature 2010; 463: 49-53.
5. Figner B, Murphy RO. Using skin conductance in judgment and decision making research. In: Schulte-Mecklenbeck M, Kuehberger A, Ranyard R, editors. A handbook of process tracing methods for decision research. New York, NY: Psychology Press: 2011. pp. 163-184.
